# Archaea Appear to Dominate the Microbiome of *Inflatella pellicula* Deep Sea Sponges

**DOI:** 10.1371/journal.pone.0084438

**Published:** 2013-12-30

**Authors:** Stephen A. Jackson, Burkhardt Flemer, Angela McCann, Jonathan Kennedy, John P. Morrissey, Fergal O’Gara, Alan D. W. Dobson

**Affiliations:** 1 Marine Biotechnology Centre, Environmental Research Institute, University College Cork, Cork, Ireland; 2 Department of Microbiology, University College Cork, Cork, Ireland; 3 BIOMERIT Research Centre, University College Cork, Cork, Ireland; University Paris South, France

## Abstract

Microbes associated with marine sponges play significant roles in host physiology. Remarkable levels of microbial diversity have been observed in sponges worldwide through both culture-dependent and culture-independent studies. Most studies have focused on the structure of the bacterial communities in sponges and have involved sponges sampled from shallow waters. Here, we used pyrosequencing of 16S rRNA genes to compare the bacterial and archaeal communities associated with two individuals of the marine sponge *Inflatella pellicula* from the deep-sea, sampled from a depth of 2,900 m, a depth which far exceeds any previous sequence-based report of sponge-associated microbial communities. Sponge-microbial communities were also compared to the microbial community in the surrounding seawater. Sponge-associated microbial communities were dominated by archaeal sequencing reads with a single archaeal OTU, comprising ∼60% and ∼72% of sequences, being observed from *Inflatella pellicula*. Archaeal sequencing reads were less abundant in seawater (∼11% of sequences). Sponge-associated microbial communities were less diverse and less even than any other sponge-microbial community investigated to date with just 210 and 273 OTUs (97% sequence identity) identified in sponges, with 4 and 6 dominant OTUs comprising ∼88% and ∼89% of sequences, respectively. Members of the candidate phyla, SAR406, NC10 and ZB3 are reported here from sponges for the first time, increasing the number of bacterial phyla or candidate divisions associated with sponges to 43. A minor cohort from both sponge samples (∼0.2% and ∼0.3% of sequences) were not classified to phylum level. A single OTU, common to both sponge individuals, dominates these unclassified reads and shares sequence homology with a sponge associated clone which itself has no known close relative and may represent a novel taxon.

## Introduction

Marine sponges (*Porifera*) are host to microbes from all domains of life; *Eukarya* [1], [2], *Archaea* [3], [4] and *Bacteria* [5]. These close and consistent associations are thought to be based on various symbiotic relationships; commensalist, mutualist [6] and parasitic [7]. Microbes are also a significant food source for marine sponges [8] which, as sessile animals, must derive their nutrition by active filter-feeding from ambient seawater.

Much research interest has focused on the bacterial associates of marine sponges since the early work of Clive Wilkinson [9] and Jean Vacelet [10] in the 1970s showed that bacteria comprise significant proportions of sponge tissues. Progressive advances in technologies in molecular biology have uncovered enormous levels of bacterial diversity inhabiting sponge tissues. Members of 40 bacterial phyla or candidate phyla [11]–[13], [40] as well as both major archaeal phyla [5] and eukaryotic microbes (fungi and diatoms) have to date been detected in sponge tissues through culture isolation [14], microscopy (TEM [10], FISH [15]) and molecular investigations: DGGE [16], RFLP [17], PCR [18] and latterly, pyrosequencing [11]–[12], [19]–[22].

Numerous sponge families, genera and species from tropical, temperate and polar waters have to date been investigated. These studies have revealed inter- and intra-species similarities and differences with apparent sponge-specific taxa [23] which despite being derived from disparate sponge species and distant biogeographic regions are more closely related to each other than to similar taxa from non-sponge habitats. Recently massively parallel pyrosequencing has enabled very detailed descriptions of sponge-associated microbial communities, generating sequence datasets which are many orders of magnitude greater than was previously possible. This has enabled the discovery of additional low abundance members of these microbial communities, as well as a more complete and accurate description of the structures and stability of the highly complex resident symbiont communities.

Few studies to date have considered the relative abundance of *Archaea* in sponge-associated microbial communities. Lee and colleagues [11] have however shown that *Archaea* comprise significant proportions (ranging from 4–28%) of the microbial communities inhabiting various individuals of three sponge species from the Red Sea. Such significant levels of *Archaea* within sponge tissues suggest that they may play a significant role in host physiology, particularly as they have previously been shown to be of ecological importance in nitrogen cycling [24]–[26].

In this study we use pyrosequencing of 16S rRNA genes to; (1) determine the structure of the microbial communities associated with two individuals of the deep-sea marine sponge *Inflatella pellicula*, (2) determine what contribution *Archaea* make to the microbial communities and (3) compare the sponge microbiota to that of the surrounding seawater.

## Materials and Methods

### Sampling

Sponges and seawater were sampled using the Irish research vessel, *RV Celtic Explorer* and the remotely operated vehicle (ROV) *Holland I*, from the Atlantic Ocean in Irish waters. Specific permission was not required, to obtain the marine sponge samples used in this study as they were collected in Irish territorial water, by an Irish research vessel, funded by the Irish government. The sponge samples do not involve endangered or protected sponge species. Two individuals of the marine sponge *Inflatella pellicula* (Class *Demospongiae*; Order *Poecilosclerida*; Suborder *Myxillina*; Family *Coelospheridae*) denoted IpA and IpB, were sampled from a single location (N54° 14′ 31″, W12° 41′ 38″), within ∼2 m of each other at a depth of 2900 m. Seawater was sampled at the same depth from the same location, directly adjacent to the sponges. Sampling took place in summer (July, 1^st^), 2010. Voucher specimens were curated at the Marine Institute, Galway, Ireland. Sponges were immediately rinsed with sterile artificial seawater, placed in sterile Ziploc bags and then frozen at −80°C until ready for use. Artificial seawater comprised 33.3 g/L Instant Ocean, (Aquarium Systems – Blacksburg, VA, USA), a defined ion and mineral formulation commonly used in aquaria. Seawater (30L) was collected at the sponge sampling site and immediately filtered through 0.2 µm membrane filters (Whatman – Austin, TX, USA) and the filters were stored in sterile tubes at −80°C until ready for use.

### Metagenomic DNA Extraction

Sponge tissues were weighed and finely ground under liquid N_2_ with a sterile mortar and pestle. The ground tissues were suspended in lysis buffer [100 mM Tris, 100 mM EDTA, 1.5 M NaCl (w/v), 1% CTAB (w/v), 2% SDS (w/v)] - adapted from Brady, 2007 [27]. Metagenomic DNA was then extracted as previously described [28]. Seawater metagenomic DNA was extracted from membrane filters using WaterMaster DNA Purification Kit (Epicentre Biotechnologies, Madison, WI, USA) according to the manufacturer’s instructions. DNA solutions were analysed by gel electrophoresis, quantified by spectrophotometry (NanoDrop ND-1000 – Wilmington, DE, USA) and then stored at −20°C.

### PCR Amplicon Library Preparation for Pyrosequencing

PCR amplicon libraries of the V5–V6 region of 16S rRNA genes were prepared from *I. pellicula* and seawater metagenomic DNAs. Universal primers U789f (5′-TAGATACCCSSGTAGTCC-3′) and U1068r (5′-CTGACGRCRGCCATGC-3′) [11], targeting both *Bacteria* and *Archaea*, were adapted for pyrosequencing by the addition of sequencing adapters and multiplex identifier (MID) sequences as per Table S1. Each 50 µl PCR reaction comprised 1X buffer, 0.2 mM dNTPs, 0.1 µM of each primer, 2U *Taq* polymerase, ∼10 ng template DNA and sdH_2_O. PCR cycle conditions comprised initial denaturation at 94°C for 5 min followed by 26 cycles of denaturation at 94°C for 30 s, annealing at 53°C for 30 s and extension at 72°C for 45 s. A final extension at 72°C for 6 min was added [11]. To minimise PCR bias three individual reactions were performed per template and equimolar amounts of PCR products from each of the three reactions were pooled for pyrosequencing. PCR products were purified using Qiagen PCR Purification Kit (Qiagen Ltd., UK) as per the manufacturer’s instructions. Barcoded samples were pooled and sequenced on GS FLX Titanium platform (454 Life Sciences) at the University of Liverpool, Centre for Genomic Research, Liverpool, UK.

### Pyrosequencing Data Analysis

Adapter sequences were removed from all pyrosequencing reads. Datasets were denoised using Acacia [29] and sequences were subsequently analysed using a custom script in the Quantitative Insights Into Microbial Ecology (QIIME: http://qiime.org) software package [30]. Sequencing reads were assigned to samples according to their barcodes. Reads with incorrect barcodes, incorrect primer sequences, average quality scores of <25, homopolymers of ≥6 and read lengths <200 bp were removed from further analysis. OTUs were picked using the default setting (97% similarity) using UCLUST. Representative sequences from each OTU were aligned using PyNAST. Chimeric reads were removed using Chimera Slayer. Taxonomic assignments were assigned by comparison to the Greengenes database (http://greengenes.lbl.gov). Rarefaction curves and diversity metrics (alpha diversity: Chao1 and Shannon indices, beta diversity: UniFrac) were generated at 97% sequence identities. Selected sequences from unclassified taxa were further investigated by BLAST searches [31] at the NCBI website (http://blast.ncbi.nlm.nih.gov/Blast.cgi). Further analysis of these sequences was performed by alignments using ClustalW and tree building by the neighbor-joining [32] and maximum likelihood methods using MEGA 5 (http://www.megasoftware.net/) [33]. Reference sequences were downloaded from the RDP database (http://rdp.cme.msu.edu/). All sequence data is publicly available on MG-RAST (ID no.s 4497995.3, 4497996.3, 4497998.3). (http://metagenomics.anl.gov/).

## Results and Discussion

### Sampling and Data Analysis

Pyrosequencing of 16S rRNA genes from *Archaea* and *Bacteria* was performed from two individuals of the deep-sea marine sponge *I. pellicula* sampled from a single location at a depth of 2900 m and also from seawater sampled at the same depth and location. The samples combined yielded 27791 raw sequencing reads of which 25051 reads were included in the final analysis after denoising and quality filtering. Sampling sponges from ∼3000 m ocean depths is not trivial, with accompanying logistical and financial considerations. Such reasoning dictated that only two individuals of the marine sponge *I. pellicula* were available from which to compare symbiotic microbial communities. Caution is required when using deep-sequencing data to describe microbial community structures. Sequencing errors can lead to overestimations of microbial diversity [34] while the 16S rRNA region targeted for amplification can also influence the analysis through primer biases. Amplicon sequence reads which cover both a variable region and a hypervariable region of the target gene have been recommended [35]. For that reason we targeted the V5–V6 region of the 16S rRNA gene. The primers used here have previously been used to successfully classify bacterial and archaeal pyrosequencing reads [11]. To further minimise any possible biases triplicate PCR reactions were performed for each sample. Although the archaeal abundances noted in sponges here, as well as the dominance in sponge communities of very few OTUs, suggest primer bias may be present, in parallel studies in our laboratory [36] employing the same primers on a range of other sponge species and seawater samples and all sequenced simultaneously on a single picotiter plate, vastly different results were observed with archaeal abundances ranging from 4%–25% in other sponges and ∼5000 OTUs found in one sponge species.

### Microbial Diversity

#### Archaea

**Figure 1:**
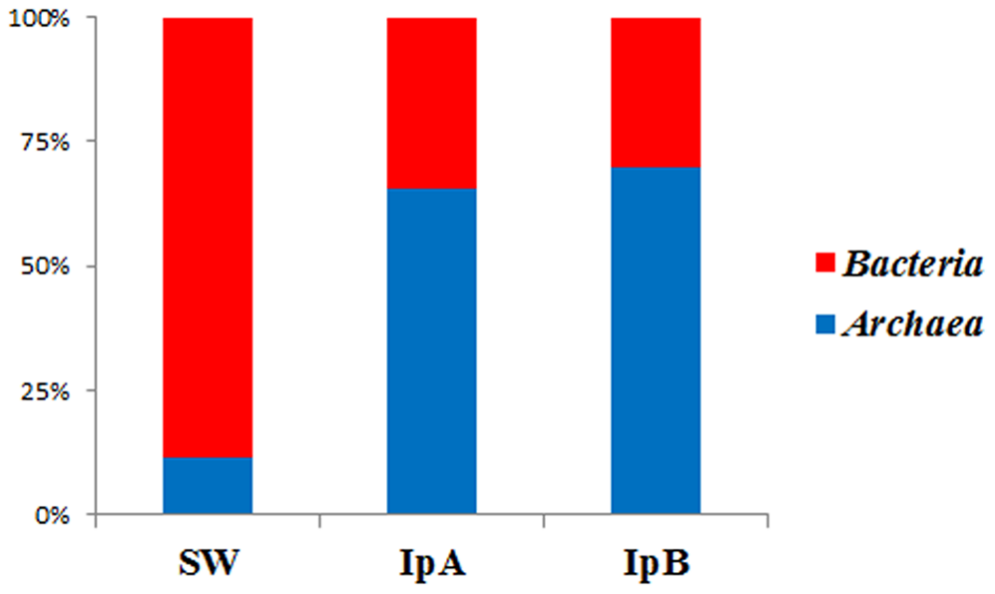
Relative Abundance by Domain. Relative abundance of 16S rRNA gene pyrosequencing reads from sponges (IpA and IpB) and from seawater classified by domain.

All seawater derived sequence reads were classified in the domains *Archaea* or *Bacteria*. From the sponges low abundance (<0.01%) sequences were not assigned to those domains. BLAST analyses revealed that the unclassified reads shared homology with sponge 18S rRNA sequences and were removed from subsequent analyses. *Archaea* comprised 61% (IpA) and 73.5% (IpB) of sponge-derived sequencing reads but only 11.3% of the seawater cohort (Figure 1).

At the phylum level, two archaeal phyla (*Euryarchaeota* and *Thaumarchaeota*) were noted in seawater. The thaumarchaeal reads from seawater (4.8% of seawater sequences) almost exclusively represent the *Cenarcheaceae* family of *Thaumarchaeota* (∼43% of seawater derived archaeal sequences). Rare seawater derived archaeal sequences (n = 6) represent Marine Benthic Group A (MBGA). The euryarchaeal sequences from seawater (6.5% of seawater reads) represent 23 OTUs (97%) from Marine group II *Archaea* in the class *Thermoplasmata* (∼47% of the seawater derived archaeal sequences) and 2 OTUs from Marine group III family of *Archaea* from the same class (∼9.9% of archaeal reads from seawater). Rare euryarchaeal reads (n = 9) represent the orders YLA114 and *Halobacteriales*. The sponge-associated *Archaea* were almost exclusively *Thaumarchaeota* in the *Cenarcheaceae* family, the exception being 0.13% of the IpB reads and 0.85% of the IpA sequences which were not classified below the phylum level. A single archaeal OTU was completely dominant in both sponge individuals. Classified in the family *Cenarcheaceae*, this OTU accounted for ∼55% of all sequencing reads from IpB and ∼69% of reads from IpA. This archaeal OTU was present at low abundance (0.07%) in seawater.

The relative abundance of archaeal reads in sponges is remarkable. *Archaea* have been known since 1996 to be associated with sponges [37]. Abundant *Archaea* have been reported in sponges previously. Sequence based studies have identified archaeal relative abundances in sponges ranging from 4%–28% of sequence reads in individual sponges (*Stylissa carteri*, *Hyrtios erectus*, *Xestospongia testudinaria* [11], *Phakiella fusca* [38]). Lee and colleagues reported 64–98% of sponge derived archaeal reads were assigned to the phylum *Crenarchaeota* while Han and co-workers reported sponge derived archaeal sequences to be exclusively *Crenarchaea*. Contrastingly, Lee reported that seawater derived archaeal reads were almost exclusively *Euryarchaeota*. Lipid biomarker analysis was used to report highly abundant (79%–90%) *Archaea* associated with the deep-sea sponge *Tenturium semisuberites* [39]. Recently, the first deep-sequencing investigation of the microbial ecology of carnivorous sponges (*Asbestopluma hypogea*) [40], revealed archaeal relative abundances of 55% and 44% in two sponge individuals. Our study however, reflects the largest archaeal abundance reported from filtering sponges, to date, in a sequence-based study. Furthermore, the archaeal communities associated with *A. hypogea* were not dominated by a single OTU as are the *I. pellicula* communities. Despite the high relative abundances of archaeal sequences in sponges here, only 15 (IpA) and 16 (IpB) archaeal OTUs were identified, of which 7 OTUs were common to both sponges. Contrastingly, the much lower abundance of archaeal sequences in seawater comprises 45 OTUs. Only 3 archaeal OTUs, were noted in sponges (both individuals) that were absent from seawater.

Many recent studies have linked the presence of *Archaea* in sponge tissues through 16S rRNA analyses to ammonia-oxidation through PCR amplification of archaeal *amoA* genes [41]–[45]. However a recent study of seven sponge species [46] revealed *amoA* genes from archaea in four species, bacterial *amoA* genes in four species and *amoA* genes from both domains present in only one species. These findings suggest largely non-redundant symbiont functions which are host selected in different species. No conclusion can be drawn however about the functional roles of the putative symbionts reported here. It is reasonable nonetheless to speculate that such a simple and dominant microbial community plays a major role in nitrogen cycling within this host, particularly as the dominant archaeal OTU is closely related to *Cenarchaeum symbiosum*. Genomic analysis of *C. symbiosum* previously revealed genes related to ammonia-oxidation, ammonia permease, and urease genes [47]. Furthermore metatranscriptome analysis of the sponge *Geodia barretti* has shown that archaeal genes predicted to be involved in ammonia-oxidation functions are among the most highly transcribed transcripts [45].

#### Bacteria

**Figure 2:**
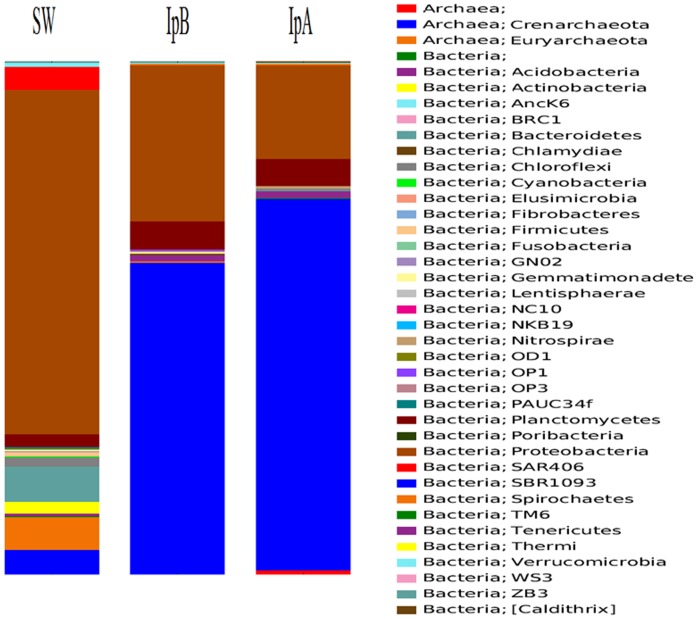
Relative Abundance by Phylum. Relative abundance of 16S rRNA gene pyrosequencing reads from sponges (IpA and IpB) and from seawater (SW) classified by phylum.

The bacterial associates of the seawater recruited to 30 bacterial phyla or candidate divisions. In contrast the sponges hosted members of 22 (IpA) and 20 (IpB) bacterial phyla or candidate phyla. Bacterial phyla common to both sponge individuals and also noted in seawater include *Acidobacteria*, *Bacteroidetes*, *Chlamydiae*, *Chloroflexi*, *Gemmatimonadetes*, NC10, NKB19, *Nitrospirae*, OD1, *Planctomycetes*, *Proteobacteria*, SAR406, SBR1093 and *Verrucomicrobia.* Phyla found in sponges but absent from seawater were *Spirochaetes*, OP1 and WS3, common to both sponge individuals, and *Caldithrix*, found in only one sponge individual (IpA). Phyla found in seawater and only one sponge individual were *Actinobacteria*, *Cyanobacteria*, GN02, OP3, TM6, *Thermi* and ZB3. Ten bacterial phyla or candidate phyla present in seawater were not found in either sponge individual (Figure 2), including the putative sponge-specific candidate phylum *Poribacteria*.

Microbial diversity was highest in seawater where 765 OTUs (97% sequence identity) were observed. Sponges hosted 210 (IpA) and 273 (IpB) OTUs (Table 1).

**Table 1:** Analysis of 16S rRNA sequencing reads from seawater and from sponges.

Sample	No. of sequencing reads	No. of archaeal phyla	No. of bacterial phyla	No. of OTUs (97% sequence identity)	Chao1 species estimator	Shannon Index
**SW**	11128	2	30	765	1206	6.24
**IpA**	5385	1	22	210	386	2.34
**IpB**	8538	2	20	273	506	2.73

Diversity indices were calculated at 97% sequence similarities. (SW = seawater; IpA = *I. pellicula* A; IpB = *I. pellicula* B).

Of the bacterial sequencing reads classified to phylum level, the most abundant phylum in sponges was *Proteobacteria* (18.2% of the IpA cohort, 30.3% of IpB sequencing reads) followed by *Planctomycetes* which comprised 5.2% of the IpA and 5.7% of the IpB communities. In contrast, *Planctomycetes* accounted for just 2.7% of seawater derived sequences while *Proteobacteria* comprised 67% of the seawater community. Apart from *Acidobacteria* which comprised 1.1% of the both sponge-associated communities, all other bacterial phyla present in sponges, were found at ≤0.2% abundances.

The relative abundances of proteobacterial sequencing reads in seawater and sponges differed greatly while the taxa and abundances in sponges were similar. Seawater hosted abundant populations of α-*Proteobacteria* (14.5% of sequences), δ-*Proteobacteria* (9% of sequences) and γ-*Proteobacteria* (42.7% of sequences). Sponges hosted minor populations of α-*Proteobacteria* [1.9% (IpB) and 1.4% (IpA) of sequences], δ-*Proteobacteria* [1.1% (IpB) and 1.3% (IpA) of sequences) and γ-*Proteobacteria* [3% (IpB) and 2.9% (IpA) of sequences]. Particularly abundant in both sponge individuals were β-*Proteobacteria* (24.5% of the IpB community, 11.6% of the IpA cohort). A single β-proteobacterial OTU from the order EC94 accounts for 99% (IpA) and 98% (IpB) of sponge-derived β-proteobacterial sequences but is absent from seawater. This enigmatic OTU is only known thus far as a coral-associated clone and has only 90% similarity to the next most closely related sequences which are from sponges and thus may be true symbionts of marine invertebrates and whose unknown function is intriguing. The proteobacterial orders and families identified in *I. pellicula* are regularly found associated with sponges [5], [11], [19], [20], including members of putative sponge-specific taxa [23]. Sequencing reads from proteobacterial taxa of notable abundance in *I. pellicula* include *Ricketsialles* (*α-Proteobacteria*), *Desulfobacterales* (*δ-Proteobacteria*), *Oceanospirillales* (*γ-Proteobacteria*) and a cohort of *γ-Proteobacteria* which were not classified below phylum level. Those unidentified taxa are closely related to cloned sequences from deep ocean waters, sediments, corals and sponges and may represent a marine clade of bacteria with no closely related cultured isolates. Members of the order *Pirellulales*, in the phylum *Planctomycetes*, comprise an abundant bacterial group in both sponge samples (∼3.1% of reads). Members of this order are ubiquitous in marine habitats, having previously been reported from marine sponges [48] and being implicated in aerobic ammonia-oxidation.

**Figure 3:**
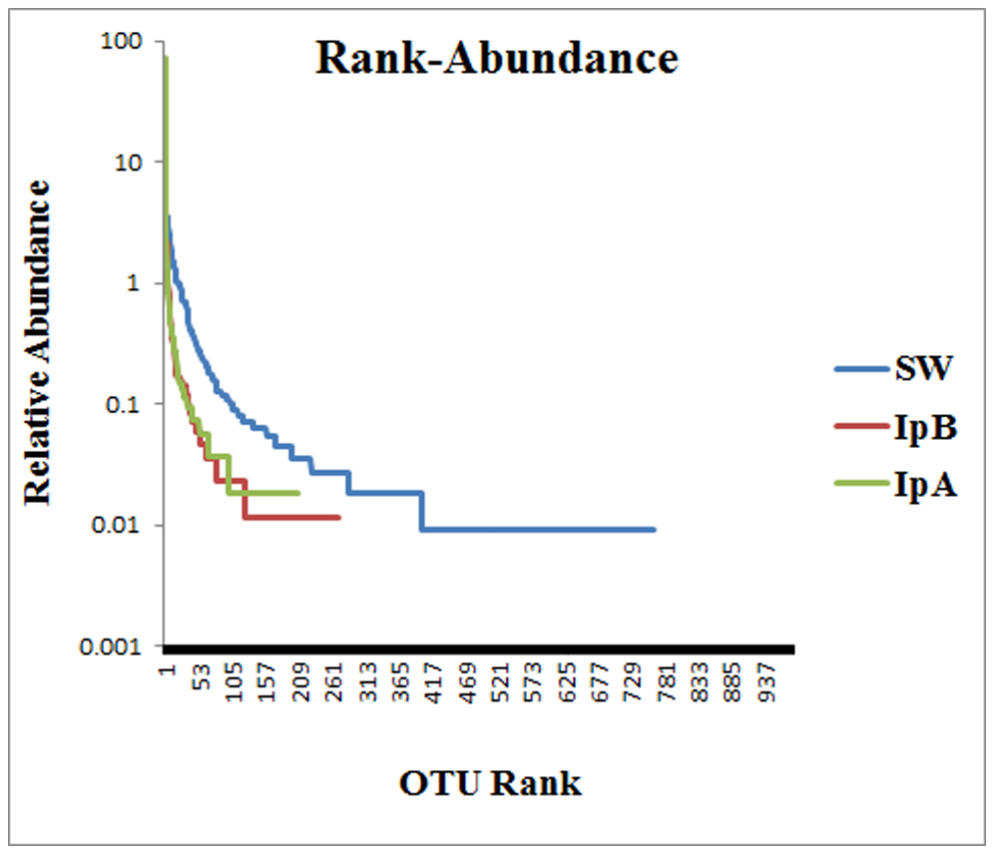
Rank-Abundance curves. Rank-Abundance curves for seawater and sponge derived pyrosequencing reads from the V5–V6 region of 16S rRNA genes from *Bacteria* and *Archaea*. OTUs are based on 97% sequence similarities. (SW = seawater; IpA = *I. pellicula* A; IpB = *I. pellicula* B).

**Figure 4:**
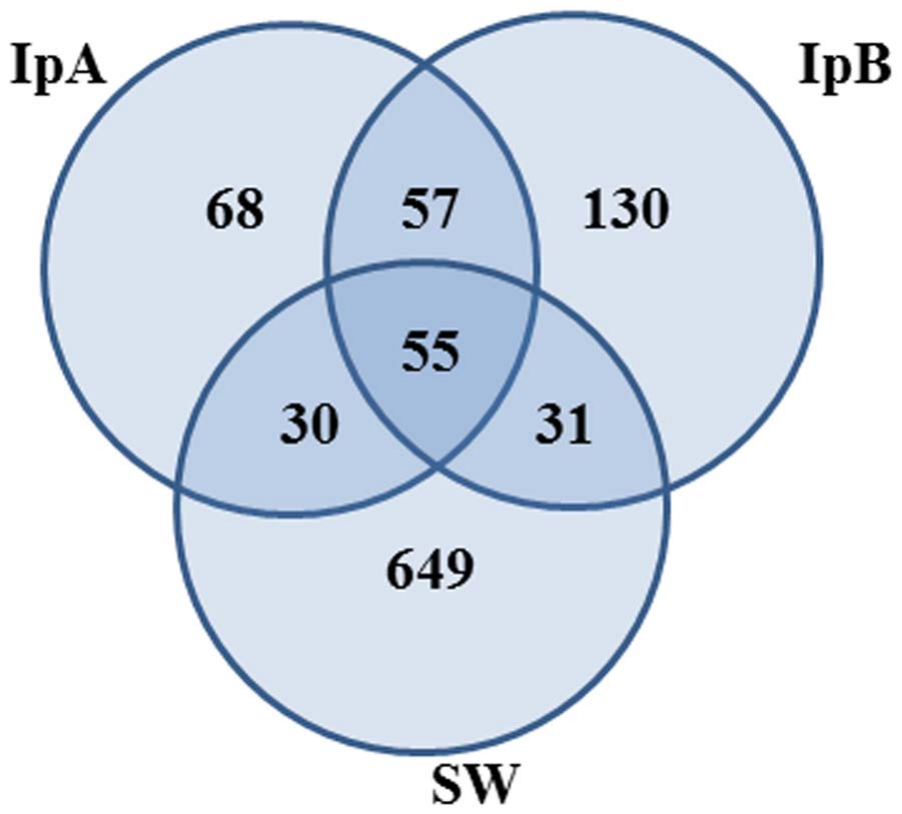
Shared OTUs. No. of OTUs (97% sequence identity) shared between sponges and seawater and no. of OTUs unique to each sample. (SW = seawater; IpA = *I. pellicula* A; IpB = *I. pellicula* B).

Very few microbial OTUs account for the major proportion of sponge-associated communities. Members of 2 archaeal OTUs together with members from 2 bacterial OTUs combined comprise ∼88% of all reads from IpA. Similarly, the same four OTUs account for ∼85% of all reads from IpB. Rank-abundance curves (Figure 3) reflect the fact that a few dominant phylotypes comprise the major proportion of the sponge communities whereas a more even community is seen in seawater. Fifty-five OTUs were common to all three communities (seawater and both sponges) while an additional 57 OTUs are present in both sponges but absent from seawater. Despite very few shared OTUs comprising the major proportion of the communities of both sponges, most individual OTUs, which were present at low abundances, were unique to one sponge sample or the other (Figure 4). Similarly, the vast majority of OTUs in seawater were not found in sponges.

### Rare Taxa

Sponge-associated microbial communities were found to host low abundance reads from members of 20 (IpA) and 19 (IpB) bacterial phyla or candidate phyla. The most abundant of these rare taxa is the phylum *Acidobacteria* which is present at ∼1.1% abundance in both sponge individuals, more than twice as abundant as in seawater (0.5%). Sequences representing the phyla *Bacteroidetes*, *Chlamydiae*, *Chloroflexi*, *Gemmatimonadetes*, *Nitrospirae*, *Spirochaetes* and *Verrucomicrobia* are present in both sponge individuals at relative abundances of ≤0.4%.

Both sponges also host low abundance (≤0.2%) reads from members of the candidate divisions NC10, NKB19, OD1, OP1, SAR406, SBR1093, and WS3. Of these candidate phyla, sequences from NC10, NKB19, OD1, SAR406 and SBR1093 are present at similarly low abundances in seawater. Low abundance reads from phyla/candidate phyla found in only one sponge individual include *Actinobacteria, Cyanobacteria*, GN02, OP3, TM6, *Thermi*, ZB3 and *Caldithrix*. WS3 and *Caldithrix* are absent from seawater while SAR406 is the third most abundant bacterial phylum (4.5% of sequences) in seawater, after *Proteobacteria* and *Bacteroidetes*. Of the candidate divisions TM6 [5], OP3, SBR1093 [12], OD1, NKB19 [13], WS3 [19] and GN02 [40] have been reported from sponges previously. However, to our knowledge, this is the first report of NC10, SAR406 and ZB3 being found in sponges. This increases the number of bacterial phyla or candidate phyla reported from sponge species worldwide to 43 (Table 2). The association of these sequencing reads to these candidate phyla was confirmed by BLAST analyses, sequence alignments and tree building (Figure S1, Table S2).

**Table 2:** Bacterial phyla and candidate divisions reported in association with marine sponges.

Phylum/Candidate phylum	Reference
*Acidobacteria, Actinobacteria, Bacteroidetes, Chloroflexi, Cyanobacteria, Deinococcus-Thermus, Firmicutes,* *Gemmatimonadetes, Lentisphaerae, Nitrospira, Planctomycetes, * ***Poribacteria*** *, Proteobacteria,* *Spirochaetes*, **TM6**, *Verrucomicrobia*	[5]
*Aquificae, Deferribacteres, Dictyoglomi*, **TM7**	[47]
BRC1, *Chlamydiae, Fibrobacteres, Fusobacteria, Tenericutes*, **WS3**	[19]
*Chlorobi, Chrysiogenetes*, **OD1**, *Thermodesulfobacteria*	[11]
*Thermotogae, Elusimicrobia, Synergistetes*	[48]
**OP3, OP10, OS-K, SBR1093**	[12]
**OP11, NKB19**	[13]
**GN02**	[40]
**NC10, SAR406, ZB3**	This study

Recognised phyla are in *italics*, candidate divisions are in **bold**.

**Figure 5:**
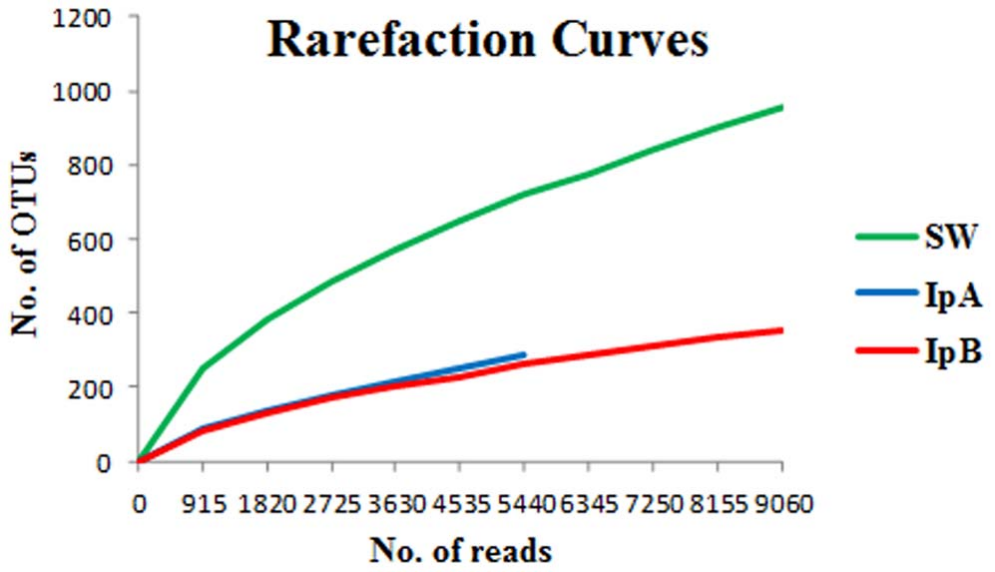
Rarefaction Curves. Rarefaction curves for seawater and sponge derived pyrosequencing reads from the V5–V6 region of 16S rRNA genes from *Bacteria* and *Archaea*. (SW = seawater; IpA = *I. pellicula* A; IpB = *I. pellicula* B). The x-axis refers to the number of sequencing reads sampled in a random sampling process.

Shannon diversity indices calculated for all three samples were reflective of the relative diversities observed (Table 1). Chao1 species estimators (Table 1) suggest that ∼54% of the sponge-associated communities and ∼63% of the seawater community have been sampled in this study. Rarefaction curves also suggest that the microbial communities were not fully represented in the data as the curves do not plateau (Figure 5). Consequently, a deeper sequencing effort would be required to assess the full microbial diversity present in these sponges. UniFrac analysis (Figure S2) confirms that the sponge-associated microbial communities are significantly different to the seawater community. This analysis not only reflects the similarity of the sponge communities (largely dominated by a few identical OTUs) but also confirms community differences (OTUs unique to one sponge or the other).

### Unidentified Taxa

A common feature of sponge-associated microbial pyrosequencing studies is the presence of bacterial taxa which cannot be assigned to phyla and are widely reported to represent possible novel taxa. Webster and colleagues identified sequence reads ranging in abundances from 0.1 to ∼16% from different sponge species and individuals which were unclassified below domain level [19]. Lee and co-workers have reported that <1% to ∼13% of sequencing reads from different sponges could not be classified to phyla [11]. White and colleagues reported that ∼24% of sequencing reads were not classifiable [21] while Schmitt *et al* [12] reported the detection of a sponge associated unclassified lineage (SAUL) in a wide range of sponges from around the world. In our study 0.003% (IpA) and 0.002% (IpB) of sponge derived bacterial reads could not be assigned to phyla. Of those sequences, one third represented an OTU common to both sponges but absent from seawater. BLAST analysis of this OTU reveals that it is closely related to other sponge derived cloned sequences which in turn are only distantly related (≤92% similar) to any other known 16S sequence. BLAST analyses and tree building were used to further investigate the phylogeny of the 14 sponge-associated OTUs not assigned to phyla. The most abundant unclassified OTU forms a monophyletic cluster with two other unclassified OTUs from the same sponge. This clade falls between the domains *Archaea* and *Bacteria* in bootstrapped phylogenetic trees (Figure S3). The closest related sequence to the most abundant unclassified OTU as revealed by BLAST search is an uncultured *Chlamydiae* cloned sequence which shares 89% sequence identity. This sequence cluster may represent a novel taxon and warrants further investigation. Phylogenetic inferences from tree-building suggests that some of the unclassified OTUs fall within the phyla *Proteobacteria*, *Firmicutes* and *Planctomycetes*, However, other unclassified OTUs appear as distinct sister branches, on monophyletic nodes, represented by *Elusimicrobia*, *Actinobacteria*, *Aquificae* and *Chloroflexi*. Although these unclassified OTUs are proximal to these phyla on the tree, they appear distinct (Figure S3) and may also represent novel taxa.

## Conclusion

The sponge *Inflatella pellicula* appears to host a unique microbial consortium. Whether the high degree of microbial community similarity observed in the two sponge individuals sampled here is reflective of the species in general or just a reflection of the particular location from which they were sampled is not yet clear, but further work on this sponge species may provide answers to that question. Pyrosequencing reads from *Archaea* present in the sponge tissues are found at very high relative abundances and the sponge communities are dominated by very few OTUs. Candidate bacterial divisions have been observed here in sponges for the first time and may be reflective of the extreme sampling location. Some dominant OTUs in the sponges are closely related to phylotypes with well-established functional capabilities which may be an important factor in the symbiotic relationship (e.g. *Thaumarchaeota*: anaerobic ammonia-oxidation, *Pirellulales*: aerobic ammonia-oxidation), while the potential metabolic capabilities of others (e.g. EC94, β*-Proteobacteria*) are as of yet unknown. Although extensive sequencing efforts in recent years have revealed enormous levels of microbial diversity in marine sponges, most samples are from shallow waters and our knowledge of sponge-microbe communities from the deep-sea is still very limited.

## Supporting Information

Figure S1 Bootstrap consensus (*n* = 1000) Maximum Likelihood phylogenetic tree illustrating the affiliation of sponge derived sequence reads with candidate phyla not previously identified in sponges. Bootstrap values, which represent the percentage of trees in which the associated taxa clustered together, are shown next to the branches. The tree is drawn to scale, with branch lengths measured in the number of substitutions per site. Sponge derived sequences are denoted with solid red circles.
(DOC)

Figure S2 Bootstrap consensus UPGMA UniFrac analysis of pyrosequencing reads from seawater (SW) and sponges (IpA & IpB).
(DOC)

Figure S3 Bootstrap consensus (*n* = 500) Maximum Likelihood phylogenetic tree illustrating the inferred phylogeny of sponge derived sequencing reads not classified to phylum level. Bootstrap values, which represent the percentage of trees in which the associated taxa clustered together, are shown next to the branches. The tree is drawn to scale, with branch lengths measured in the number of substitutions per site. Sponge derived sequences are shaded in grey boxes and illustrate the inferred taxonomic positions of the sponge-derived OTUs.
(DOC)

Table S1 Primer design for pyrosequencing of 16S rRNA (V5–V6) genes from *Archaea* and *Bacteria* in sponges and seawater.
(DOC)

Table S2 Closest BLAST relatives to unclassified sponge OTUs.
(DOC)
