# Supplementary material for: Archaea Appear to Dominate the Microbiome of Inflatella pellicula Deep Sea Sponges
Source: PLoS One. 2013 Dec 30;8(12):e84438. doi: 10.1371/journal.pone.0084438 (PMC3875569; doi:10.1371/journal.pone.0084438)
Supplement: Figure S1 — Bootstrap consensus (n = 1000) Maximum Likelihood phylogenetic tree illustrating the affiliation of sponge derived sequence reads with candidate phyla not previously identified in sponges. Bootstrap values, which represent the percentage of trees in which the associated taxa clustered together, are shown next to the branches. The tree is drawn to scale, with branch lengths measured in the number of substitutions per site. Sponge derived sequences are denoted with solid red circles. (DOC) [file pone.0084438.s001.doc]

**Figure S1**: Bootstrap consensus (*n*=1000) Maximum Likelihood phylogenetic tree illustrating the affiliation of sponge derived sequence reads with candidate phyla not previously identified in sponges. Bootstrap values, which represent the percentage of trees in which the associated taxa clustered together, are shown next to the branches. The tree is drawn to scale, with branch lengths measured in the number of substitutions per site. Sponge derived sequences are denoted in red with solid red circles.
