# Supplementary material for: Archaea Appear to Dominate the Microbiome of Inflatella pellicula Deep Sea Sponges
Source: PLoS One. 2013 Dec 30;8(12):e84438. doi: 10.1371/journal.pone.0084438 (PMC3875569; doi:10.1371/journal.pone.0084438)
Supplement: Figure S2 — Bootstrap consensus UPGMA UniFrac analysis of pyrosequencing reads from seawater (SW) and sponges (IpA & IpB). (DOC) [file pone.0084438.s002.doc]

**Figure S2**: Bootstrap consensus UPGMA UniFrac analysis of pyrosequencing reads from seawater (SW) and sponges (IpA & IpB).
