# Supplementary material for: Archaea Appear to Dominate the Microbiome of Inflatella pellicula Deep Sea Sponges
Source: PLoS One. 2013 Dec 30;8(12):e84438. doi: 10.1371/journal.pone.0084438 (PMC3875569; doi:10.1371/journal.pone.0084438)
Supplement: Figure S3 — Bootstrap consensus (n = 500) Maximum Likelihood phylogenetic tree illustrating the inferred phylogeny of sponge derived sequencing reads not classified to phylum level. Bootstrap values, which represent the percentage of trees in which the associated taxa clustered together, are shown next to the branches. The tree is drawn to scale, with branch lengths measured in the number of substitutions per site. Sponge derived sequences are shaded in grey boxes and illustrate the inferred taxonomic positions of the sponge-derived OTUs. (DOC) [file pone.0084438.s003.doc]

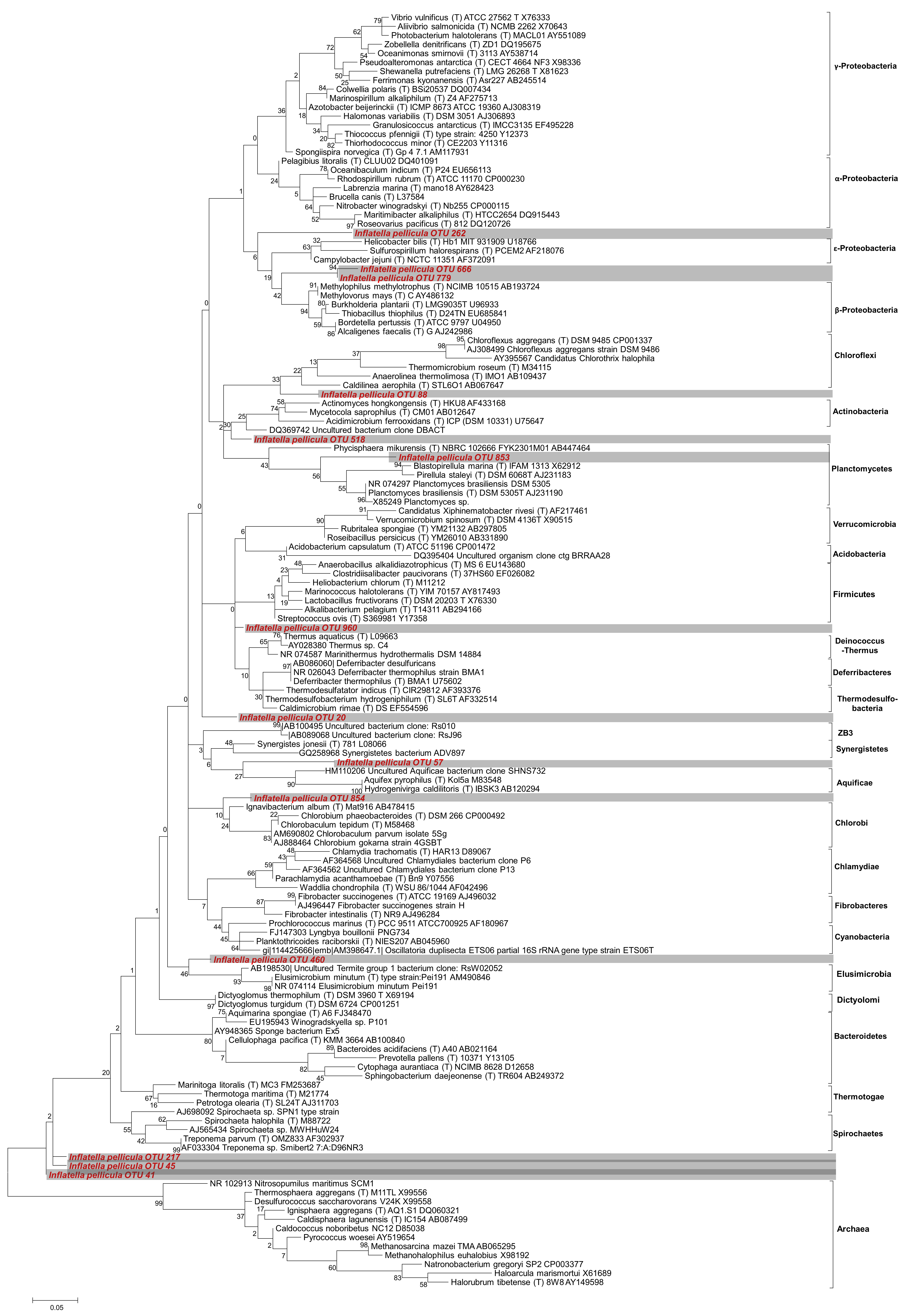


**Figure S3**: Bootstrap consensus (*n*=500) Maximum Likelihood phylogenetic tree illustrating the inferred phylogeny of sponge derived sequencing reads not classified to phylum level. Bootstrap values, which represent the percentage of trees in which the associated taxa clustered together, are shown next to the branches. The tree is drawn to scale, with branch lengths measured in the number of substitutions per site. Sponge derived sequences are shaded in grey boxes and illustrate the inferred taxonomic positions of the sponge-derived OTUs.
