# Supplementary material for: Archaea Appear to Dominate the Microbiome of Inflatella pellicula Deep Sea Sponges
Source: PLoS One. 2013 Dec 30;8(12):e84438. doi: 10.1371/journal.pone.0084438 (PMC3875569; doi:10.1371/journal.pone.0084438)
Supplement: Table S1 — Primer design for pyrosequencing of 16S rRNA (V5–V6) genes from Archaea and Bacteria in sponges and seawater. (DOC) [file pone.0084438.s004.doc]

| **Sample** | **Primer** | **Adapter** | **Multiplex Identifier (MID)** | **Template specific primer** |
| --- | --- | --- | --- | --- |
| **SW**  **(seawater)** | Forward | CGTATCGCCTCCCTCGCGCCATCAG | ACGCTCGACA | TAGATACCCSSGTAGTCC (U789F) |
|  | Reverse | CTATGCGCCTTGCCAGCCCGCTCAG | ACGCTCGACA | CTGACGRCRGCCATGC (U1068r) |
| **IpA**  **(*I.pellicula* A)** | Forward | CGTATCGCCTCCCTCGCGCCATCAG | TCTCTATGCG | TAGATACCCSSGTAGTCC (U789F) |
|  | Reverse | CTATGCGCCTTGCCAGCCCGCTCAG | TCTCTATGCG | CTGACGRCRGCCATGC (U1068r) |
| **IpB**  **(*I.pellicula* B)** | Forward | CGTATCGCCTCCCTCGCGCCATCAG | TGATACGTCT | TAGATACCCSSGTAGTCC (U789f) |
|  | Reverse | CTATGCGCCTTGCCAGCCCGCTCAG | TGATACGTCT | CTGACGRCRGCCATGC (U1068r) |

**Table S1**: Primer design for pyrosequencing of 16S rRNA (V5-V6) genes from *Archaea* and *Bacteria* in sponges and seawater.
