# Supplementary material for: Archaea Appear to Dominate the Microbiome of Inflatella pellicula Deep Sea Sponges
Source: PLoS One. 2013 Dec 30;8(12):e84438. doi: 10.1371/journal.pone.0084438 (PMC3875569; doi:10.1371/journal.pone.0084438)
Supplement: Table S2 — Closest BLAST relatives to unclassified sponge OTUs. (DOC) [file pone.0084438.s005.doc]

**Table S2**: Closest BLAST relatives to unclassified sponge OTUs.

| **OTU ID** | **TOP BLAST HIT** | **NCBI Accession No.** | **% SEQUENCE IDENTITY** | **PHYLOGENY INFERRED FROM TREE** |
| --- | --- | --- | --- | --- |
| OTU20 | Syntrophomonas lipocalidus DSM 12680 (Firmicutes) | NC_014220 | 94 | ? |
| OTU41 | Chlamydophila psittaci 6BC (Chlamydiae) | NC_015470 | 89 | ? |
| OTU45 | Borrelia miyamotoi LB-2001 (Spirochaetes) | NC_022079 | 93 | ? |
| OTU57 | Dehalogenimonas lykanthroporepellens BL-DC-9 (Chloroflexi) | NC_014314 | 94 | ? |
| OTU88 | Spiribacter salinus M19-40 (Proteobacteria) | NC_021291 | 89 | ? |
| OTU217 | Treponema succinifaciens DSM 2489 (Spirochaetes) | NC_015385 | 91 | ? |
| OTU262 | Thalassolituus oleivorans MIL-1(Proteobacteria) | NC_020888 | 88 | Proteobacteria |
| OTU460 | Desulfobacca acetoxidans DSM 11109 (Proteobacteria) | NC_015388 | 90 | ? |
| OTU518 | Syntrophomonas wolfei subsp. wolfei str. Goettingen (Firmicutes) | NC_008346 | 93 | ? |
| OTU666 | Desulfovibrio alaskensis G20 (Proteobacteria) | NC_007159 | 89 | Proteobacteria |
| OTU779 | Candidatus Puniceispirillum marinum IMCC1322 (Proteobacteria) | NC_014010 | 90 | Proteobacteria |
| OTU853 | Candidatus Methylomirabilis oxyfera (NC10) | NC_013260 | 86 | Planctomycetes |
| OTU854 | Thioalkalivibrio sp. K90mix (Proteobacteria) | NC_013889 | 92 | ? |
| OTU960 | Syntrophomonas lipocalidus DSM 12680 (Firmicutes) | NC_014220 | 94 | ? |
